# Fc-GDF15 glyco-engineering and receptor binding affinity optimization for body weight regulation

**DOI:** 10.1038/s41598-021-87959-5

**Published:** 2021-04-26

**Authors:** Ella Fung, Liya Kang, Diana Sapashnik, Susan Benard, Annette Sievers, Yan Liu, Guoying Yan, Jing Zhou, Linette Rodriguez, Weijun Ma, Wayne R. Stochaj, Edward LaVallie, Liliana Wroblewska, Kerry Kelleher, Amy Tam, Olivier Bezy, Danna Breen, Jeffrey R. Chabot, Tao He, Laura Lin, Zhidan Wu, Lidia Mosyak

**Affiliations:** 1 BioMedicine Design, Pfizer Inc., 610 N Main Street, Cambridge, MA USA; 2 Internal Medicine Research Unit, Pfizer Inc., 1 Portland Street, Cambridge, MA USA; 3 Present Address: Sanofi Research and Development, Sanofi North America, Framingham, MA USA; 4 Present Address: Cellarity, Cambridge, MA USA; 5 Present Address: JOINN Biologics US Inc, Richmond, CA USA

**Keywords:** Protein design, Recombinant protein therapy

## Abstract

GDF15 is a distant TGF-β family member that induces anorexia and weight loss. Due to its function, GDF15 has attracted attention as a potential therapeutic for the treatment of obesity and its associated metabolic diseases. However, the pharmacokinetic and physicochemical properties of GDF15 present several challenges for its development as a therapeutic, including a short half-life, high aggregation propensity, and protease susceptibility in serum. Here, we report the design, characterization and optimization of GDF15 in an Fc-fusion protein format with improved therapeutic properties. Using a structure-based engineering approach, we combined knob-into-hole Fc technology and N-linked glycosylation site mutagenesis for half-life extension, improved solubility and protease resistance. In addition, we identified a set of mutations at the receptor binding site of GDF15 that show increased GFRAL binding affinity and led to significant half-life extension. We also identified a single point mutation that increases p-ERK signaling activity and results in improved weight loss efficacy in vivo. Taken together, our findings allowed us to develop GDF15 in a new therapeutic format that demonstrates better efficacy and potential for improved manufacturability.

## Introduction

In recent years, GDF15 has come to light as a powerful regulator of appetite and body weight. It has long been known that circulating GDF15 levels correlate with lower BMI and cachexia in patients with cancer, heart failure, or chronic kidney disease^1–3^. Recently, understanding of the mechanism of action has evolved: circulating GDF15 binds its receptor GFRAL, which is selectively expressed in the area postrema (AP) and nuclear solitary tract (NTS) in the hindbrain, where it signals through a co-receptor RET^4–7^. Current evidence suggests that the activation of GFRAL-expressing neurons stimulates neurons in the parabrachial nucleus and central amygdala, resulting in appetite suppression and ultimately body weight loss. The GDF15-GFRAL-RET signaling pathway is well conserved in rodents and non-human primates.

The function of GDF15 as an appetite suppressor has raised the possibility of pharmacologically administering GDF15 to reduce body weight^8^. Several key pieces of in vivo data support this notion. Firstly, transgenic mice overexpressing GDF15 from birth were protected from diet-induced obesity, hepatic steatosis, and glucose intolerance^1,9^. Secondly, GDF15 administration through either viral vectors or recombinant protein injection in a genetic obesity ob/ob mouse model reduced food intake, body weight, and improved overall metabolic parameters such as glucose tolerance and insulin sensitivity^10,11^. Lastly, these benefits were reproducible in obese non-human primates dosed weekly with Fc-GDF15, strengthening confidence in the therapeutic potential of GDF15^11^. Taken together, these data support GDF15 as an intervention for obesity and its associated metabolic disorders.

However, the pharmacokinetic and physicochemical properties of wildtype GDF15 present several key challenges for its development as a therapeutic. Its half-life is extremely short, at 3 h in mice and non-human primates^11^, which is undesirable for chronic conditions as it demands frequent dosing. GDF15 also has a high aggregation propensity resulting in low stability and expression titer. In vivo, extracellular GDF15 undergoes proteolytic cleavage making it unstable in serum, thus presenting little value as a therapeutic^12^.

One approach which has been used to improve the production profile and half-life of GDF15 is Fc-fusion^11^. It is well established that fusion to an Fc domain can extend protein half-life via neonatal Fc receptor (FcRn) recycling^13–16^. Indeed, approved Fc-fusion biotherapeutics currently on the market have a half-life of between 4 and 16 days in the case of etanercept and abatacept respectively^17^. However, for multimeric proteins such as GDF15, Fc-fusion frequently leads to daisy-chaining and aggregation during production, severely impacting titer and yield. One engineering solution to prevent such multimerization is by pairing an Fc-GDF15 arm with a stump Fc arm, for example using charged-pair mutations or a single-chain Fc^11^. Here, we utilize knob-into-hole Fc technology^18^. Mutations in the Fc variants that drive heterodimerization without compromising biophysical and functional properties such as conformational stability and FcRn binding^19^ have been also reported for GDF15 analogs in patent literature^20^.

A second approach to improve the physicochemical properties of GDF15 is glycan engineering^21^. N-linked glycans (N-glycans) are highly soluble, branched molecules ranging from approximately 1.5–2.5 kDa in size. The addition of N-glycans to target proteins can reduce aggregation propensity by shielding hydrophobic patches, resulting in a tenfold improvement in activity in the case of an IFN-β therapeutic (Refib^®^)^22^. This strategy for increasing solubility of GDF15 has also been explored in patent literature^20^. Additionally, N-glycans can be designed to shield protease cleavage sites on the target protein, a strategy we used to enhance the protease resistance of an FGF21 variant^23^. Here, we apply glycan masking to GDF15 protease cleavage sites for the first time. Glycan engineering also offers an opportunity to extend GDF15 half-life, as glycans containing sialic acid are associated with longer circulating lifetimes^21^. This was seen for a hyperglycosylated erythropoietin (darbepoetin alfa, Aranesp^®^) where increased sialic acid content tripled half-life^24^.

Using a structure-based, rational design approach, we combine knob-into-hole Fc technology with glycan engineering to improve the half-life and solubility of GDF15. We then further optimize the receptor binding affinity of our GDF15 variant using site-directed mutagenesis, enhancing its weight loss efficacy and further doubling half-life in vivo.

## Results

### Fc-fusion and N-glycans improve the production profile of GDF15

We first developed an Fc-fusion strategy. The crystal structure of the mature GDF15 dimer bound to GFRAL (PDB 5VZ4) shows that the C-terminal isoleucine of each monomer is packed against a hydrophobic patch of the opposite monomer^6^. To avoid disrupting dimer formation, we introduced the Fc-fusion at the N-terminal of GDF15. Furthermore, the co-receptor RET is known to complex with other GDNF/GFRα family members using a composite binding site, interacting with both the ligand and co-receptor. This was recently confirmed for the GDF15-GFRAL-RET ternary complex using cryo-EM^25^, which showed an interaction site for RET on the concave face of GDF15. To avoid steric hindrance of RET, the Fc domain was separated from GDF15 by a flexible 25 amino acid linker (G_4_S)_5_.

**Figure 1:**
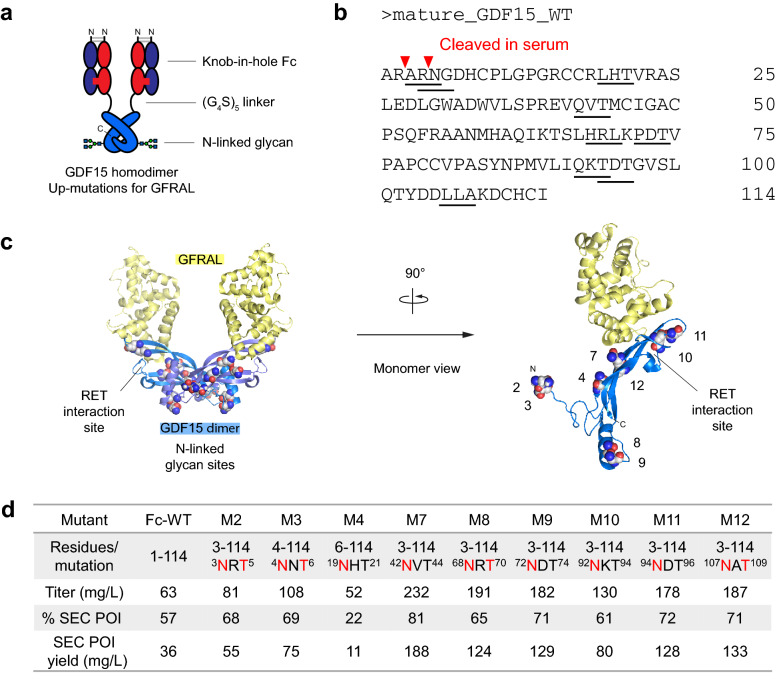
Fc fusion and N-linked glycans improve the production profile of GDF15. (**a**) Schematic representation of proposed Fc-glyco GDF15 therapeutic. Red, Knob-Fc chain. Blue, Hole-Fc chain. Light blue, mature GDF15 homodimer. N and C termini are highlighted. (**b**) Mature GDF15 amino acid sequence (UniProt Q99988, PRO_0000033993). Sites to introduce N-linked glycosylation are underlined. Red, putative serum cleavage sites. (**c**) Location of introduced N-glycan sites modeled on the GDF15-GFRAL crystal structure (PDB 5VZ4) using The PyMOL Molecular Graphics System, Version 2.0 Schrödinger, LLC (https://pymol.org/). Introduced asparagine (N) mutations are shown as spheres. Numbers correspond to glyco-variant number (eg. Mutant 2). (**d**) Summary of Fc-GDF15 NxT mutant expression and purification yields, including expression titer (mg/L), Protein of Interest (POI) percentage following size exclusion chromatography (SEC), and SEC yield.

By applying this strategy, we designed GDF15 in an Fc-fusion format as shown in Fig. 1a. Each GDF15 monomer was fused to a knob-Fc at its N-terminal via a flexible linker. The knob-Fc-GDF15 paired with a co-expressed hole-Fc, a stump arm. The knob-in-hole Fc prevented the formation of “daisy-chain” aggregates by limiting the binding of each Fc-GDF15 arm to a single other Fc-GDF15 arm. Other Fc formats were also tested, including an IgG Fc-GDF15 homodimer and a hole-Fc-GDF15/knob-Fc-GDF15 heterodimer, however both had high levels of aggregation and chain mispairing (data not shown). We refer to knob-in-hole Fc as simply Fc from here onwards unless otherwise stated.

Next, we selected sites to introduce N-linked glycosylation. This can be achieved in a highly site-specific manner by introducing NxT motifs to the GDF15 peptide sequence. During protein expression, NxT sites naturally undergo glycosylation at the ER lumen and Golgi apparatus. Using the GDF15-GFRAL crystal structure as a guide, glycosylation sites were introduced to produce a panel of single NxT mutants (Fig. 1b, Supplementary Fig. 1). We aimed to avoid the GFRAL receptor binding site to preserve GDF15 signaling activity (Fig. 1c). We note that at the time of modeling, the structure of GDF15-GFRAL bound to RET was not available. Two introduced N-linked glycosylation sites, Mutants 10 and 11, were located near the site of RET interaction (Fig. 1c).

In addition, N-glycan engineering offered an opportunity to improve the serum stability of Fc-GDF15. The annotated mature GDF15 sequence is 114 amino acids in length (UniProt Q99988, PRO_0000033993), with mature sequence numbering 1–114 to which we will refer throughout the paper (Fig. 1b). GDF15 is well reported to undergo cleavage by PCSK family proteases, which recognize the motif Rx_2n_R, where n = 0–3^26^. The mature GDF15 sequence contains two cleavage sites: one well-characterized site at R2 giving a 112 amino-acid GDF15 peptide^27,28^, and a putative cleavage site at R4 (Fig. 1b). Importantly, extracellular cleavage of GDF15 at the N-terminal has been observed in vivo^12^, therefore an N-terminal Fc-fusion has a potential disadvantage of being clipped in circulation. To address this, we explored N-glycan masking around protease sensitive sequences. We first excluded the R2 cleavage site by deletion of the first two residues and introduced an N-glycan site to mask the putative R4 cleavage site by an A3N/N5T substitution (Fig. 1d, Mutant 2). We also tested the R4 site using an R3N/G5T mutation (Fig. 1d, Mutant 3). The remainder of the NxT panel contained glycans introduced further downstream in the GDF15 sequence, with the first R2 serum cleavage site excluded.

These mutants were expressed using the transient Expi293 cell system and purified using Protein A affinity capture and size exclusion chromatography. During production, we observed that the addition of an Fc-fusion dramatically improved the titer of GDF15 to 63 mg/L (Fig. 1d), compared to native GDF15 expression levels of < 1 mg/L. The addition of an N-glycan further improved titer, up to 230 mg/L for Mutant 7. We also observed an improvement in the percentage protein of interest (POI) with reduced aggregation during size exclusion chromatography (Fig. 1d). The exception to this was Mutant 4, which showed high levels of aggregation. This variant excluded the first five amino acids of the mature GDF15 sequence, which may contribute to correct protein folding and stability. Due to its low POI yield, it was dropped from characterization studies.

### N-glycans improve serum stability with minimal impact on GDF15 activity

**Figure 2:**
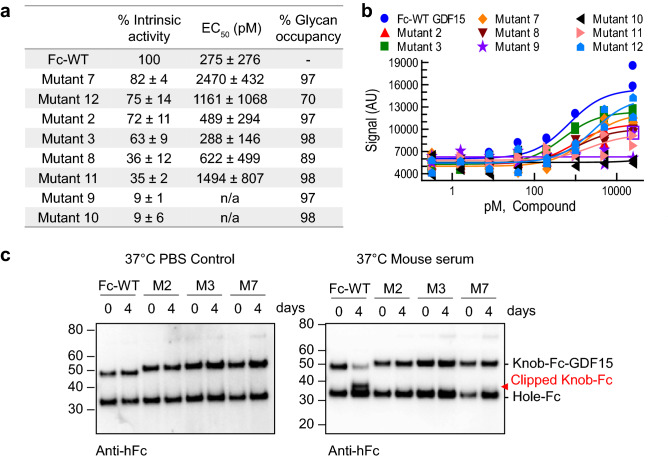
N-glycans improve serum stability with minimal impact on GDF15 activity. (**a**) Summary of Fc-GDF15 functional activity and glycan occupancy, from highest intrinsic activity to lowest. Functional activity was measured by Phospho-ERK HTRF (Homogeneous Time Resolved Fluorescence) assay and reported as EC_50_ and % intrinsic activity (maximum response, Ymax) normalized to wildtype Fc-GDF15 (Fc-WT). Data are reported as mean ± SD. N = 4. EC_50_ values of “n/a” indicate that EC_50_ could not be determined due to low activity. Glycan occupancy at engineered NxT sites was measured using capillary gel electrophoresis. (**b**) Representative data from p-ERK assay summarized in (**a**). N = 4. The Y-axis values represent the HTRF signal ratio (665/615 nm × 10^4^). (**c**) In vitro serum stability of wildtype Fc-GDF15 control (Fc-WT), Mutant 2, Mutant 3, and Mutant 7 following 4 days of incubation in mouse serum or a PBS control at 37 °C. Gross clipping was detected by Western blot using an anti-human Fc antibody. Clipped chain highlighted in red. Full length blots are presented in Supplementary Fig. 6.

Next, the purified Fc-GDF15 variants were functionally validated using a homogeneous time-resolved fluorescence (HTRF) phospho-ERK assay. CHO cells stably expressing GFRAL and RET were treated with Fc-GDF15 glyco-variants, or a wildtype Fc-GDF15 control. Phosphorylated ERK (p-ERK) levels were reported as normalized intrinsic activity (% of maximum response from Fc-GDF15, Ymax) and EC_50_ (Fig. 2a,b). The data showed that the wildtype Fc-GDF15 control was most efficacious, and that the introduction of N-glycans reduced activity to various degrees in all screened locations (Fig. 2a). Some variants had only minor reductions in functional activity, such as Mutant 7 (85 ± 4% Ymax), whereas Mutants 9 and 10 were completely inactive. While our N-glycans were designed to avoid obstruction of the GFRAL binding site, modeling using the recently published structure of the GDF15-GFRAL-RET ternary complex^25^ revealed that engineered N-glycans may interfere with RET binding and signal transduction (see “Discussion”).

Despite reductions in signaling activity, we anticipated that our Fc-glyco variants would be more serum stable compared to the wildtype control as a result of deletion and potential glycan masking of the R2 and R4 cleavage sites respectively. We therefore selected the three variants that had the highest intrinsic signaling activity relative to the wildtype control: Mutant 7 (85 ± 4% Ymax), Mutant 2 (72 ± 11% Ymax), and Mutant 3 (63 ± 9% Ymax). These three mutants also had > 97% glycan occupancy as determined by capillary gel electrophoresis, demonstrating that these functional data were not confounded by non-glycosylated protein product (Fig. 2a, Supplementary Fig. 2). Despite having high intrinsic activity, Mutant 12 was deselected due to low glycan occupancy (70%).

We next assessed in vitro serum stability. Our three lead Fc-GDF15 glyco-variants (Mutants 2, 3 and 7) or a wildtype Fc-GDF15 control were incubated in mouse serum for 4 days at 37 °C. Gross clipping was then detected by Western blot (Fig. 2c). We observed that the wildtype Fc-GDF15 control was least serum stable, with 82% of material clipped by Day 4. This produced a ~ 35 kDa knob-Fc peptide, consistent with N-terminal cleavage of GDF15. This process was entirely serum-dependent, as a control sample incubated in PBS was stable (Fig. 2c). In contrast to the wildtype Fc-GDF15, no clipping was observed in Mutants 2 and 3 in which cleavage site R4 was expected to be shielded by the new N-glycans. Consistent with this, Mutant 7 with cleavage site R4 exposed showed low-level clipping. Based on these observations, we surmised that elimination and masking of cleavage sites R2 and R4 respectively both contributed to the superior in vitro serum stability of Mutants 2 and 3. For this reason, Mutants 2 and 3 were selected as top leads for further optimization.

### Screening point mutants for increased receptor binding

**Figure 3:**
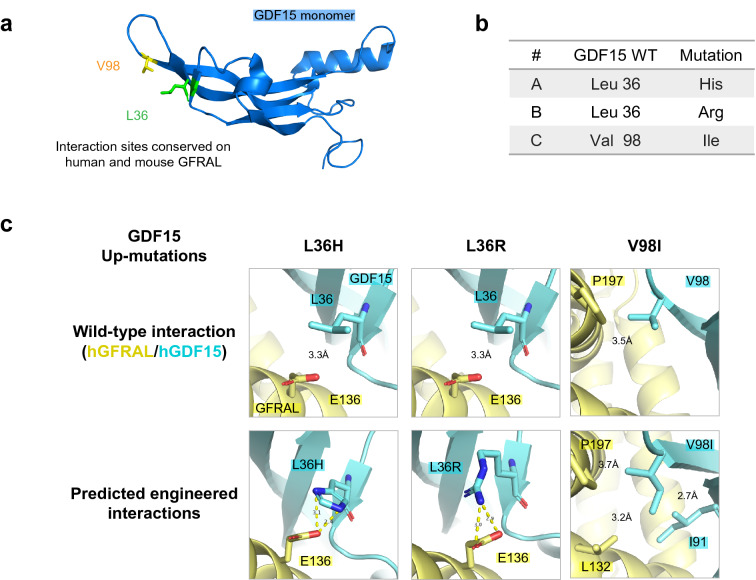
Introduction of point mutants to improve GFRAL binding affinity. (**a**) Structure of GDF15 monomer. Highlighted residues at the GFRAL binding site were selected for mutation (PDB 5VZ4). (**b**) GDF15 point mutations designed to improve GFRAL binding affinity. (**c**) Predicted engineered interactions modeled on GDF15-GFRAL crystal structure (PDB 5VZ4). Wildtype interaction shown above. Theoretical up-mutant interactions shown below. Blue, hGDF15. Yellow, hGFRAL. Yellow dashed lines denote predicted hydrogen bonding interactions. Figures rendered using The PyMOL Molecular Graphics System, Version 2.0 Schrödinger, LLC (https://pymol.org/).

The crystal structure for the GDF15-GFRAL complex (PDB 5VZ4)^6^ also offered the opportunity to design point mutations to increase the binding affinity of GDF15 for GFRAL (up-mutations). Suitable positions for mutations were estimated based on the conservation of binding residues in both human and mouse GFRAL, allowing us to interrogate their effect in animal studies. To assist the selection of engineered single-point mutations we used a sequence tolerance prediction algorithm that identifies substitutions as tolerated without compromising a protein interface, while maintaining a desired function^29^. Our final set of selected sequences for further evaluation includes three up-mutations as shown in Fig. 3, annotated A-C. Mutants A (L36H) and B (L36R) were each designed to introduce hydrogen bonding interactions, and Mutant C (V98I) was designed to fill a hydrophobic void at the binding interface. These up-mutations were individually introduced to the NxT backgrounds of Mutants 2 and 3 to give a panel of Fc-GDF15 up-mutants, each containing one N-glycan and one up-mutation (2A, 2B, 2C etc.). We predicted that these new up-mutations could increase the signaling activity of Mutants 2 and 3, while building upon their improved production profiles and serum stability. The panel of up-mutants was produced as described above apart from Mutant 2B, which did not express in Expi293 and was dropped from further study.

Surface plasmon resonance (SPR) was used to measure the binding affinity of our Fc-GDF15 up-mutants for human and mouse GFRAL. The wildtype Fc-GDF15 control had a binding affinity of 11 nM for hGFRAL (Table 1, Supplementary Tables 1, 2). Mutants 2 and 3 had comparable receptor binding affinity to the wildtype (10 nM and 11.2 nM respectively). This confirmed that the introduced N-glycans did not affect GFRAL binding. Based on this, we concluded that the impact of N-glycans on p-ERK signaling activity (Fig. 2a) was likely due to effects on RET binding rather than on GFRAL (see “Discussion”).

**Table 1:** Screening up-mutants for GFRAL binding affinity and p-ERK signaling activity.

Fc-GDF15 variant	NxT mutation	Up-mutation	K_D_ analysis (N = 2)		Functional activity (N = 3)	
			hGFRAL ECD K_D_ (nM)	mGFRAL ECD K_D_ (nM)	% Intrinsic activity (Ymax)	EC_50_ (pM)
Fc-GDF15 WT	–	–	11.0 ± 8.2E−10	230.0 ± 4.2E−08	100	177 ± 95
Mutant 2	A3N/N5T	–	10.0 ± 4.0E−11	245.0 ± 3.3E−08	64 ± 17	114 ± 19
Mutant 2A	A3N/N5T	L36H	6.03 ± 2.3E−11	186.0 ± 1.3E−08	66 ± 18	312 ± 61
Mutant 2C	A3N/N5T	V98I	5.52 ± 4.0E−10	95.8 ± 3.2E−09	85 ± 16	436 ± 277
Mutant 3	R4N/G6T	–	11.2 ± 3.7E−10	300.0 ± 8.1E−08	59 ± 18	298 ± 95
Mutant 3A	R4N/G6T	L36H	6.09 ± 4.5E−12	178.0 ± 2.8E−09	67 ± 15	4838 ± 2165
Mutant 3B	R4N/G6T	L36R	3.68 ± 8.9E−11	82.8 ± 2.0E−09	47 ± 14	4225 ± 3773
Mutant 3C	R4N/G6T	V98I	5.44 ± 4.0E−11	124.0 ± 4.2E−08	45 ± 12	642 ± 210

Table summarizing in vitro characteristics of Fc-GDF15 up-mutants compared to a wildtype (WT) Fc-GDF15 control. Binding affinity (K_D_) was measured for recombinant human and mouse GFRAL extra-cellular domain (ECD) using surface plasmon resonance (N = 2). Functional activity was measured using p-ERK HTRF assay and reported as EC_50_ and % intrinsic activity (Ymax) normalized to Fc-GDF15 WT (N = 3). Data are shown as mean ± SD.

SPR data also showed that the introduction of up-mutation B (L36R) to Mutant 3 improved binding affinity threefold for both human and mouse GFRAL (Table 1, Mutant 3 vs Mutant 3B). Mutant 3 had a K_D_ of 11.2 nM for hGFRAL whereas Mutant 3B had a K_D_ of 3.7 nM. Other up-mutations did not significantly improve binding affinity of either Mutant 2 or 3.

We next asked whether up-mutations improved the functional activity of Mutants 2 and 3 using the p-ERK signaling assay (Table 1). As before, the wildtype Fc-GDF15 control was most active. Relative to wildtype control, Mutants 2 and 3 had 64 ± 17% and 59 ± 18% Ymax respectively. The addition of up-mutation C (V98I) to Mutant 2 substantially improved its function, increasing its activity to 85 ± 16% Ymax (*P* = 0.053, paired t-test). This was surprising, as Mutant 2C had a modest twofold improvement in GFRAL K_D_. The introduction of up-mutation C to Mutant 3 did not have a similar improvement in activity (Mutant 3C, 45 ± 12% Ymax). Other up-mutations did not improve the functional activity of Mutant 2 or 3. We note that the best performing up-mutant by SPR, Mutant 3B, did not see a corresponding increase in functional activity (47 ± 14% Ymax), suggesting there wasn’t a clear correlation between binding affinity and functional activity as demonstrated by these mutations (see “Discussion”). We also produced and screened combinations of two up-mutations, however these did not further enhance functional activity (data not shown).

### Fc-GDF15 mutant 2C (A3N/N5T/V98I) has improved weight loss efficacy

Despite the higher functional activity of wildtype Fc-GDF15 in vitro, we hypothesized that the Fc-glyco variants would be more stable and therefore more efficacious in vivo due to their improved serum stability (Fig. 2c). Four leads were selected for further investigation: Mutants 2 and 3, the glyco-variants with the highest serum stability and functional activity, and Mutants 2C and 3B, the up-mutants with the highest p-ERK signaling activity and GFRAL binding affinity respectively. In vivo grade material was produced for all four leads and a wildtype Fc-GDF15 control using stable CHO cell lines and purified as described above.

**Figure 4:**
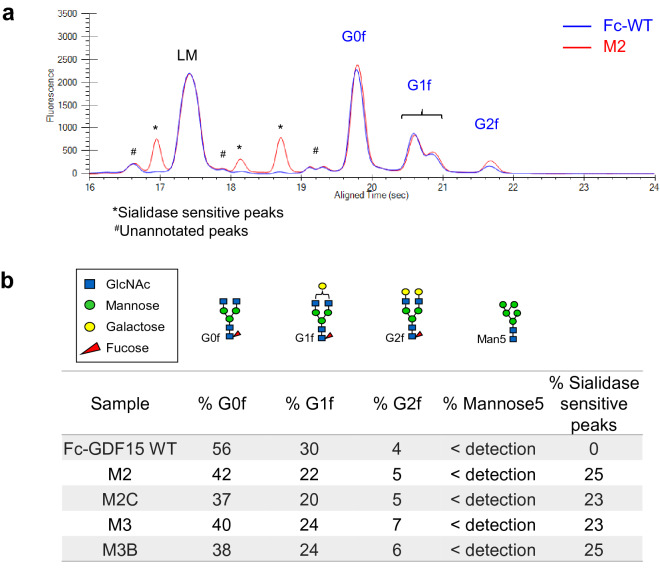
Fc-GDF15 glyco variants contain sialidase sensitive glycans. (**a**) Representative glycan profiling trace using capillary gel electrophoresis, comparing wildtype Fc-GDF15 control (Fc-WT, blue) and Mutant 2 (red). LM, lower marker. Asterisks denote sialidase sensitive peaks. Pound symbols denote unannotated peaks that did not align to glycan standards. (**b**) Summary table of major identified glycan species for Fc-GDF15 WT control and variants.

Glycoprotein structures are known to affect protein behaviors in vivo such as circulating half-life, clearance, and bioavailability^21^, therefore prior to in vivo studies we performed glycan profiling using capillary gel electrophoresis (Fig. 4). We observed that the wildtype Fc-GDF15 control primarily had G0f and G1f glycans, belonging to the Fc domain (Fig. 4a). The four Fc-glyco variants shared similar levels of G0f and G1f glycans to control and had additional species of sialidase sensitive glycans belonging to GDF15 (Fig. 4b, Supplementary Fig. 3), which are associated with longer circulation half-life^21^. Mannose-5 species, which are rapidly cleared in vivo^30^, were below detection limit for all four leads. Glycan occupancy at the engineered site was above 98% in all cases (Supplementary Fig. 3).

To test the weight loss efficacy of our Fc-GDF15 variants in vivo, we injected a single subcutaneous dose of our four leads and the wildtype Fc-GDF15 control in wildtype mice and measured daily body weight, food intake, and terminal serum Fc-GDF15 concentrations. The study duration was limited to 14 days to avoid immunogenicity, as our variants contained human Fc and GDF15 sequences. To control for potency differences and probe the effects of stability alone, animals were dosed at 0.5 mg/kg to reach maximal efficacy (Supplementary Fig. 4).

**Figure 5:**
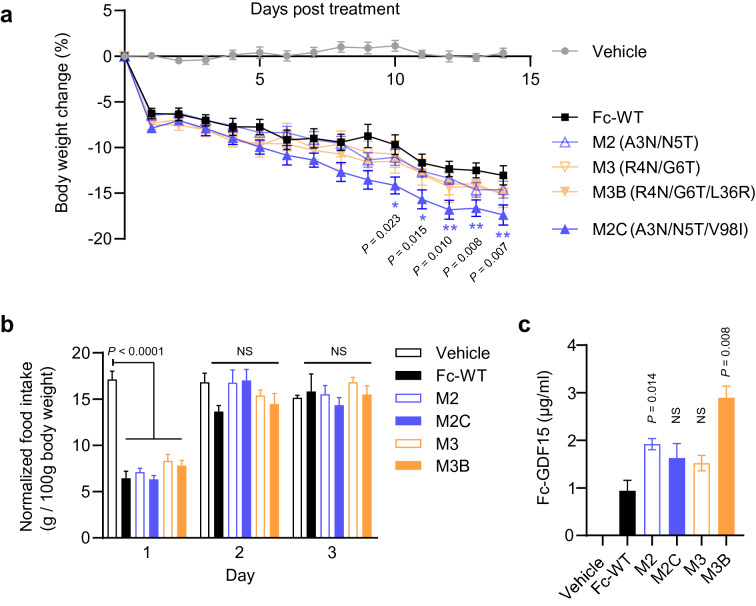
Fc-GDF15 Mutant 2C (A3N/N5T/V98I) has improved weight loss efficacy in vivo. (**a**) Body weight change after a single subcutaneous dose of the indicated Fc-GDF15 variant or the wildtype Fc-GDF15 (Fc-WT) control. Dose, 0.5 mg/kg. n = 7–10 per group. Data are reported as mean ± SEM *P ≤ 0.05,  **P ≤ 0.01 for Fc-GDF15 WT treatment as compared to all other treatments. Mixed effects model followed by Tukey’s post hoc test. (**b**) Daily food intake normalized to body weight following Fc-GDF15 treatment in (**a**). Data are reported as mean ± SEM; NS, *P* > 0.05. Mixed effects model followed by Tukey’s post hoc test. (**c**) Serum concentration of Fc-GDF15 variants on Day 14. Data are reported as mean ± SEM and analyzed with a pairwise Wilcoxon test. NS, *P* > 0.05.

Following a single dose of Fc-GDF15, we observed rapid and dramatic body weight loss accompanied by 1 day of reduced food intake (Fig. 5a,b). Initial body weight loss from Days 1–9 was comparable between all Fc-GDF15 variants, as expected from dosing at Emax. From Day 10 onwards, differences in treatment groups began to emerge, consistent with serum stability differences between Fc-GDF15 variants. Animals treated with Mutant 2C had significantly lower body weight compared to wildtype Fc-GDF15 treated animals (Fig. 5a). Other leads remained comparable to the wildtype Fc-GDF15 control, consistent with Mutant 2C having higher functional activity in vitro (Table 1, 85 ± 16% Ymax).

Terminal serum analysis revealed that Mutant 2C exposure levels were 1.6 ± 0.31 µg/ml (n = 10), trending higher than wildtype Fc-GDF15 levels at 0.9 ± 0.16 µg/ml (n = 7), however this difference did not reach significance. Importantly, our ELISA assay detected only intact Fc-GDF15 molecules by using an anti-GDF15 capture antibody and anti-hFc detection antibody. Other, less active leads had comparable exposure to Mutant 2C, suggesting that its weight loss efficacy is driven primarily by enhanced activity rather than exposure. For example, Mutant 2 and Mutant 2C had comparable exposure, however Mutant 2 had substantially lower p-ERK signaling activity (Table 1, 64 ± 17% Ymax) and comparable weight loss efficacy to Fc-WT.

Unexpectedly, Mutant 3B exposure was significantly higher than all other leads at 2.9 ± 0.24 µg/ml (n = 10). Pharmacokinetic analysis revealed that its exposure was driven by a longer circulating half-life, which was nearly double that of the wildtype Fc-GDF15 control, and not by increased bioavailability (Supplementary Fig. 5). Despite this, Mutant 3B had only average weight loss efficacy due to its low functional activity (Table 1, 47 ± 14% Ymax).

In sum, the identification of N-glycan Mutant 2 (A3N/N5T) combined with up-mutation C (V98I) allowed us to produce an Fc-GDF15 variant with substantial improvements in production profile, protease resistance, and functional efficacy in vivo. Similarly, the combination of N-glycan Mutant 3 (R4N/G6T) with up-mutation B (L36R) resulted in a second variant with substantially improved production profile, protease resistance, and half-life extension in vivo.

## Discussion

GDF15 is a potent driver of anorexia and body weight loss, and has been proposed as a therapeutic for obesity and its associated metabolic diseases^8^. Recent studies have demonstrated that GDF15 produces a durable weight loss phenotype for over a year in ob/ob mice under an adeno-associated virus overexpression system^11^, highlighting its potential as an obesity therapeutic with durable weight loss. It has also been reported that GDF15-mediated weight loss is not only dependent on food intake, but is partially dependent on a peripheral sympathetic nervous system hypothesized to regulate lipid oxidation^31^. While these findings were specific to the cancer cachexia setting, they suggest that there may be mechanistic differentiation to a GDF15-based therapeutic unlike classic anorectic agents such as GLP-1.

Here, we extend existing Fc-GDF15 engineering efforts through a combination of glycan engineering and receptor binding optimization, in order to address three engineering goals: improved production profile, efficacy, and pharmacokinetics of GDF15.

The introduction of a knob-into-hole Fc fusion dramatically improved production profiles while minimizing multimerization, as observed by Xiong et al. using charge-paired Fc and single-chain Fc technologies^11^. Against this background, glycan engineering further improved physicochemical properties. The introduction of N-glycans to Fc-GDF15 further increased expression yields, solubility, and titers, while simultaneously increasing sialic acid content for potential half-life extension. We also took advantage of glycan engineering to address GDF15 protease susceptibility at R2 and putative R4 cleavage sites. Importantly, we showed that mutants with an exposed, putative R4 site had low-level clipping compared to those masked by a glycan, demonstrating that it is a bona fide cleavage site. This was mitigated through glycan engineering in current work, thus we believe that our Fc-glyco format is more protease resistant.

While our engineered glycans were designed to be unobtrusive, we observed reduced p-ERK signaling activity for all glyco mutants compared to the wildtype control. This trend was not reflected in SPR data, which measured the 1-to-1 binding of Fc-GDF15 to GFRAL, suggesting that the N-glycans affected RET binding and higher order complex formation.

Indeed, the recently published ternary structure of GDF15-GFRAL-RET offered an explanation for the observed impact of our NxT mutations^25^. Our modeling results showed that Q92N, a mutation abolishing activity in Mutant 10 and T94N, a mutation reducing activity in Mutant 11 are both located at the GDF15-RET interaction site (Supplementary Fig. 7), explaining their adverse effects on activity. Other glycans affecting activity assume locations outside of the GDF15-RET interaction site resolved in the structure. Mutations P72N in inactive Mutant 9 and H68N in reduced activity Mutant 8 are both at the membrane proximal face of GDF15 (Supplementary Fig. 7), close to the region where the un-resolved Linker Domain of RET is predicted to reside. This domain is proposed to affect RET dimerization, transphosphorylation, and signal transduction^24^. The remaining NxT mutations that are not expected to obstruct GDF15-RET interaction showed comparatively minor losses in activity. Considering the small size of GDF15, it is perhaps unsurprising that the addition of a bulky glycan structure could have a minor deleterious effect on protein conformation and exert propagated effects on activity.

This unexpected caveat of glycan engineering was addressed through a second round of engineering. We identified a point mutation (V98I) that, when introduced to Mutant 2, compensated for its reduced signaling activity, resulting in a variant with improved production profile and serum stability without compromising function. Indeed, Mutant 2C was most efficacious in vivo (Fig. 5). This improvement was accompanied by a twofold improvement in GFRAL binding affinity, which may favor the subsequent recruitment and activation of RET. We note that improvements in GFRAL binding affinity did not necessarily lead to functional improvements for all up-mutants (Table 1), although the reason for this is not evident. One potential explanation could be differences in RET recruitment to these variants, however further investigation in the form of RET binding studies would be required to confirm this hypothesis.

A surprising observation was that the introduction of point mutation L36R to Mutant 3 led to a threefold improvement in GFRAL binding affinity, longer circulating lifetime, and significantly higher exposure (Fig. 5c, Mutant 3B). Theoretically, tighter receptor binding could extend half-life by generating a localized Fc-GDF15 reservoir in vivo. However, the high stabilization in exposure levels observed here (µg/ml range) are disproportionate to the small population of GFRAL-positive neurons described in the brainstem^4–7^. Given the size of this GFRAL-positive population, we would anticipate durable half-life extension but at lower levels. One hypothesis is that GDF15 may have additional binding partners or receptors elsewhere. A potential binding partner could be the murine splice variant GRAL-B, which encodes a soluble GFRAL lacking the transmembrane domain^32^. To our knowledge, evidence for the protein product of the GRAL-B transcript has not yet been described. Further investigation would be required to confirm this hypothesis and is beyond the scope of our study.

It is worth noting that nausea and emesis are legitimate concerns for a GDF15-based obesity therapeutic. Animal studies have had mixed results; on one hand, it has been shown that GDF15 induces malaise in mice and rats, and emesis in musk shrews^33,34^. On the other hand, clinical monitoring of non-human primates found that therapeutic doses of GDF15 did not induce nausea, malaise, or emesis^5^. These side-effects may also be dose-dependent, as the lowest efficacious dose of GDF15 did not trigger emesis in musk shrews^34^. In such cases, the prevalence of adverse effects can be reduced by using dose-titration studies to determine a therapeutic window. This was done for a GLP-1R agonist, an anti-diabetic drug known to cause anorexia in some patients^35,36^. Ultimately, clinical studies would be required to determine whether nausea is contraindicative for GDF15 as an obesity therapeutic. We propose that our Fc-GDF15 Mutants 2C and M3B would be advantageous for such applications due to their improved efficacy and half-life respectively, as well as their improved manufacturability profiles. Further studies using dose response models and in the form of animal obesity models would be required for the selection of a clinical candidate from these early leads.

In conclusion, engineering of TGF-β cytokines remains challenging given their high propensity for aggregation, pro-domain processing, and co-receptor regulation. While GDF15 drug variants exist in the intellectual property space, none of the existing patent literature has described the impact of such engineering concept on the physicochemical and pharmacokinetic properties of GDF15 to the extent that we have. We believe that this study will be of great interest to scientific community, especially to those interested in developing therapeutics for TGF-β family cytokines and involved pathologies.

## Materials and methods

### Cell culture, stable and transient expression

Mammalian cell lines were grown and maintained in a humidified incubator with 5% or 8% CO_2_ at 37 °C. Expi293 cells (Thermo Fisher) were grown in FreeStyle 293 Expression Medium (Thermo Fisher). CHOK1SV cells (Lonza) stably overexpressing Fc-GDF15 variants were grown in CD CHO Medium (Thermo Fisher). CHO-K1 cells (ATCC) stably overexpressing GFRAL/RET were grown in DMEM/F12 Medium (Gibco) supplemented with 10% FBS (Gibco).

For transient transfection in Expi293 cells (Thermo Fisher), DNA was incubated with sterile 25 kDa linear polyethylenimine (PEI) at a 1:1.5 ratio for 30 min in Opti-MEM I Reduced Serum Medium (Thermo Fisher). The DNA-PEI solution was then added to Expi293 cells seeded at a density of 3 × 10^6^ cells/ml. 4 h later, cells were treated with 0.5% tryptone N1 and 4 mM valporic acid. Following 5 days of expression, conditioned media was harvested by pelleting cells at 3000 g for 10 min before sterile filtering using a 0.2 μm PES filter.

CHOK1SV (Lonza) cell lines stably expressing Fc-GDF15 variants were generated using a site-specific integration system as previously described^37^.

Dual human GFRAL and RET stable co-expression cell line was generated by transfection of two individual plasmids containing GFRAL or RET cDNA into CHO-K1 cells (ATCC). The transfected cells were selected with antibiotics. Individual clonal cells were tested for gene expression and responses to GDF15 stimulation. A clone with the best activity was chosen as the GFRAL and RET co-expression cell line.

### Expression and purification of Fc-GDF15

Fc-GDF15 variants were cloned into a pRY19 vector compatible with our site-specific integration platform^37^. Knob-Fc-GDF15 chains and hole-Fc chains containing effector function null mutations^38^ were cloned into a single vector, each under a hCMV promoter and murine IgG VH signal sequence for secretion. Point mutants were generated using site-directed mutagenesis.

Fc-GDF15 variants were transiently expressed in Expi293 as described above and purified using an automated ÄKTA pure (GE Healthcare) chromatographic system. Briefly, conditioned media was first applied over a MabSelect SuRe column (GE Healthcare) equilibrated in PBS pH 7.2, and eluted with 100% step 150 mM Glycine, 40 mM NaCl, pH 3.5. The eluate was immediately neutralized with 1 M Tris pH 8. Eluate was then concentrated to 20 mg/ml using a 30 kDa MWCO centrifugal device and purified using size exclusion chromatography (SEC) by applying over a Superdex 200 column (GE Healthcare) equilibrated in PBS. Peak fraction purity was analyzed using a YMC Pack Diol-200 column (YMC), and fractions with greater than 97% purity were pooled. Before use, final pools were tested for endotoxin levels using a Limulus amebocyte lysate (LAL)-based Endosafe nexgen-PTS system (Charles River Laboratories), and for Protein A levels using a Protein A ELISA (Cygnus Technologies).

In vivo grade material was produced in stable CHO cell lines stably expressing Fc-GDF15 variants. The established CHO pools were subject to a 12-day fed-batch platform expression process in CD CHO media. Conditioned media was then processed and Fc-GDF15 purified as described above. Cell lines used for production were tested for mycoplasma and Mouse/Rat pathogens using the Mouse/RAT Comprehensive CLEAR panel (Charles River Laboratories).

### Expression and purification of recombinant GFRAL extracellular domain

Soluble human or mouse GFRAL extracellular domain (ECD) were cloned into a pTT5 vector with a monomeric Fc^39^ and thrombin cleavage site introduced at the N-terminus. ECD sequences cloned were residues 19–351 for human GFRAL, and residues 19–350 for mouse GFRAL. Constructs were expressed in Expi293 cells as described above. Conditioned media was batch bound to mAb Select SuRe Resin (GE Healthcare) for 1.5–2 h at 4 °C. Bound resin was packed into a chromatography column and washed with 7 CV of PBS before eluting with a 20 CV gradient from PBS to 150 mM Glycine pH 3.5, 40 mM NaCl. Eluate was immediately neutralized with 1 M Tris pH 8. Fractions containing MonoFc-GFRAL were further purified using a Superdex 200 column (GE Healthcare) equilibrated in PBS. Finally, the MonoFc tag was cleaved with bovine thrombin (Sigma) at a ratio of 1 unit of thrombin per 100 μg of protein for 2 h at room temperature. The thrombin was then removed with Soybean Trypsin inhibitor resin. The cleaved MonoFc was removed with Mab Select Sure resin.

### Glycan occupancy and profiling

Glycan analysis was performed using the LabChip GXII Touch protein characterization system (PerkinElmer). To determine glycan occupancy of engineered N-link sites, samples were processed using a HT Protein Express Reagent Kit (PerkinElmer) according to manufacturer instructions. Deglycosylated controls were generated using PNGase F (NEB). For glycan profiling, samples were processed using the ProfilerPro Glycan Profiling Assay Kit (PerkinElmer). Briefly, samples were reduced, digested with PNGase F to release N-glycans, and the N-glycans fluorescently labeled. Glycans were identified using a panel of commercially available N-glycan standards (QA-Bio). Sialidase-sensitive peaks were identified using negative controls treated with Sialidase A (Prozyme).

### In vitro serum stability

Fc-GDF15 variants were incubated at 5 μM in either PBS, pH 7.2 or mouse serum (Sigma) for 96 h at 37 °C, with time points taken at 0, 48, and 96 h. Gross clipping was detected using Western blot using a cross-adsorbed anti-Human IgG Fc (Thermo Fisher).

### Phospho-ERK HTRF functional assay

Fc-GDF15 variants were functionally validated in CHO-K1 cells stably co-expressing RET and GFRAL. Briefly, 6000 cells/well were seeded in 384-well plates in culture medium and maintained in a 37 °C incubator overnight. Various concentrations of Fc-GDF15 were directly added to the cells for a 10-min stimulation at room temperature. The media was then discarded, and the cells lysed. The phosphorylated ERK in the cell lysates was detected with the Advanced Phospho-ERK Kit (64AERPEH, CISBIO) according to manufacturer instructions. The emission signals at 665 nM and 615 nM were captured using an EnVision Multilabel Plate Reader (PerkinElmer). The 665/615 nM ratio data were analyzed by a four-parameter logistic curve fitting model using XLfit software.

### GFRAL binding affinity (K_D_) measurements

Binding affinity was determined using the Biacore T200 (Cytiva) at 37 °C. A biosensor surface was prepared by immobilizing anti-human IgG (Cytiva BR-1008-39) on a CM4 chip (Cytiva BR-1005-34) using amine coupling according to the manufacturer’s instructions. GDF15 variants fused to human Fc (Fc-GDF15) were captured on the surface. Two-fold dilution series of human and murine GFRAL ranging in concentration from 25 to 1.56 nM for human and 100 nM to 6.25 nM for murine were injected for 60 s and followed for a dissociation period of 120 s. Data were processed using Biacore T200 Evaluation Software V 3.2 (Cytiva). After double referencing, kinetic analysis was performed using a 1:1 Langmuir binding model. The result for each Fc-GDF15 variant was reported as k_on_ (on-rate), k_off_ (off-rate) and K_D_ (equilibrium dissociation constant) for both analytes. Each interaction was investigated in duplicate.

### Animal studies

Animal experiments were carried out in accordance with relevant guidelines and regulations following study protocols and procedures reviewed and approved by Pfizer Institutional Animal Care and Use Committee. Experiments were reported following ARRIVE guidelines. C57BL/6N mice were obtained from Taconic Biocsiences. All experiments were performed using lean, male, 10–12 week old animals. Animals were singly housed in temperature- and humidity-controlled rooms with a 12 h light/dark cycle, maintained on standard chow, and had free access to food and water. Prior to the study start, animals were randomized such that each group had comparable body weights. Body weight and food intake were measured within a 2-h window at the beginning of each light cycle. Fc-GDF15 was diluted in saline solution (5 mM Acetate salt, 240 mM Propylene Glycol and 0.007% Polysorbate 20, pH 4) for subcutaneous administration. Food intake was normalized by the body weight of each animal and reported as food intake per 100 g body weight.

### Fc-GDF15 ELISA

Circulating Fc-GDF15 concentrations were measured using an in-house immunosorbent assay. Fc-GDF15 was immobilized using an anti-GDF15 antibody (R&D Systems) and detected with a biotin-labeled anti-hFc antibody (Southern Biotech) and DELFIA time-resolved-fluorescence (TRF) reagents (PerkinElmer). Emission was detected using an EnVision Multimodal Plate Reader (PerkinElmer) using manufacturer recommended DELFIA instrument settings. Data were interpolated using a four-parameter logistic standard curve using GraphPad Prism software.

### PK studies and modeling

Pharmacokinetics of wildtype Fc-GDF15 and Fc-GDF15 Mutant 3B were evaluated in male C57BL/6N mice at 0.1 mg/kg following a single subcutaneous injection. Serum samples were collected at the indicated time points and stored at -80 °C until used for analysis. Serum levels of Fc-GDF15 variants were determined using an in-house Fc-GDF15 ELISA described above.

The terminal half-life of the various GDF15 constructs was determined by fitting a single exponential decay to the measurements collected from day 7 to day 14 (to avoid interference from the distribution phase). Fits were performed in GraphPad Prism 8.1 (GraphPad Software, Inc.). To test for significant differences in half life, a comparison between decay rates was performed using an extra sum-of-squares F test with a significance cutoff of *P* = 0.05, constraining the plateau of both curves to 0 and with unshared initial values.

## Supplementary Information

Supplementary Information.
