# Supplementary material for: Fc-GDF15 glyco-engineering and receptor binding affinity optimization for body weight regulation
Source: Sci Rep. 2021 Apr 26;11:8921. doi: 10.1038/s41598-021-87959-5 (PMC8076310; doi:10.1038/s41598-021-87959-5)
Supplement: Supplementary file 1 — Supplementary Information. [file 41598_2021_87959_MOESM1_ESM.pdf]

Supplementary Information for

Fc-GDF15 glyco-engineering and receptor binding affinity optimization for body weight regulation

Ella Fung, Liya Kang, Diana Sapashnik, Susan Benard, Annette Sievers, Yan Liu, Guoying Yan, Jing Zhou, Linette Rodriguez, Weijun Ma, Wayne R Stochaj, Edward LaVallie, Liliana Wroblewska, Kerry Kelleher, Amy Tam, Olivier Bezy, Danna Breen, Jeffrey R Chabot, Tao He, Laura Lin, Zhidan Wu, Lidia Mosyak*

*Corresponding author. Email: Lidia.Mosyak@pfizer.com

The PDF file includes:

Supplementary Table 1. Supplementary table of ka, kd, and KD measurements for Fc-GDF15 mutants binding human GFRAL extracellular domains 1-3 (D1D2D3).

Supplementary Table 2. Supplementary table of ka, kd, and KD measurements for Fc-GDF15 mutants binding mouse GFRAL extracellular domains 1-3 (D1D2D3).

Supplementary Figure 1. Location of glyco mutants on mature GDF15 dimer.

Supplementary Figure 2. Representative capillary gel electrophoresis traces to determine glycan occupancy.

Supplementary Figure 3. Glycan analysis of in vivo Fc-GDF15 material.

Supplementary Figure 4. Dose-finding study.

Supplementary Figure 5. Mutant 3B (R4N/G6T/L36R) has extended half-life compared to wildtype Fc-GDF15.

Supplementary Figure 6. Full scans of Western blots in Figure 2c.

Supplementary Figure 7. Location of engineered N-glycan sites in GDF15-GFRAL-RET ternary structure.

Supplementary Table 1. Supplementary table of ka, kd, and KD measurements for Fc-GDF15 mutants binding human GFRAL extracellular domains 1-3 (D1D2D3).

Ligand	Analyte	ka (1/Ms)	kd (1/s)	KD (nM)
Fc-GDF15 WT	hGFRAL D1D2D3	1.07E+07	1.20E-01	11.0
Fc-GDF15 Mut 2	hGFRAL D1D2D3	1.00E+07	1.00E-01	10.0
Fc-GDF15 2A	hGFRAL D1D2D3	6.47E+06	3.90E-02	6.03
Fc-GDF15 2C	hGFRAL D1D2D3	1.03E+07	5.74E-02	5.52
Fc-GDF15 Mut 3	hGFRAL D1D2D3	1.00E+07	1.13E-01	11.2
Fc-GDF15 3A	hGFRAL D1D2D3	6.61E+06	4.02E-02	6.09
Fc-GDF15 3B	hGFRAL D1D2D3	7.72E+06	2.83E-02	3.68
Fc-GDF15 3C	hGFRAL D1D2D3	1.05E+07	5.71E-02	5.44

Supplementary Table 2. Supplementary table of ka, kd, and KD measurements for Fc-GDF15 mutants binding mouse GFRAL extracellular domains 1-3 (D1D2D3).

Ligand	Analyte	ka (1/Ms)	kd (1/s)	KD (nM)
Fc-GDF15 WT	muGFRAL D1D2D3	1.61E+06	3.64E-01	230.0
Fc-GDF15 Mut 2	muGFRAL D1D2D3	1.52E+06	3.72E-01	245.0
Fc-GDF15 2A	muGFRAL D1D2D3	8.65E+05	1.60E-01	186.0
Fc-GDF15 2C	muGFRAL D1D2D3	1.88E+06	1.79E-01	95.8
Fc-GDF15 Mut 3	muGFRAL D1D2D3	1.59E+06	4.74E-01	300.0
Fc-GDF15 3A	muGFRAL D1D2D3	1.13E+06	2.00E-01	178.0
Fc-GDF15 3B	muGFRAL D1D2D3	1.26E+06	1.05E-01	82.8
Fc-GDF15 3C	muGFRAL D1D2D3	1.69E+06	1.88E-01	124.0

Supplementary Fig. 1

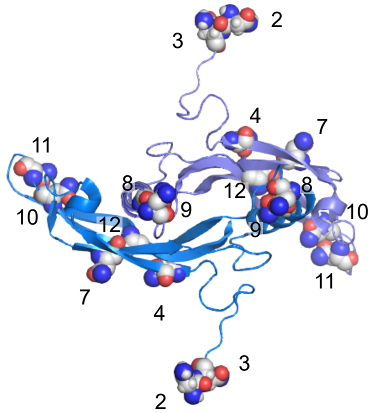

Supplementary Fig. 1. Location of glyco mutants on mature GDF15 dimer. Location of engineered N-glycan sites on the GDF15 dimer (PDB 5VZ4). Introduced asparagine (N) mutations are shown as spheres. Numbers correspond to glyco-variant number (eg. Mutant 2). Figure rendered using The PyMOL Molecular Graphics System, Version 2.0 Schrödinger, LLC (https://pymol.org/).

Supplementary Fig. 2

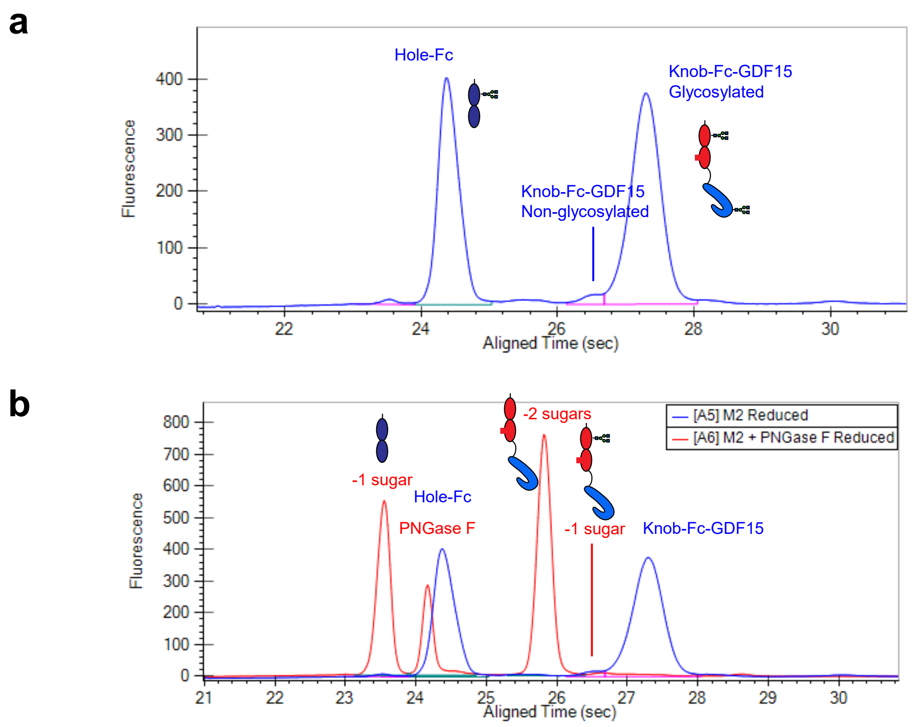

Supplementary Fig. 2. Representative capillary gel electrophoresis traces to determine glycan occupancy. (**a**) Sample trace of reduced Fc-GDF15 Mutant 2 showing two main peaks as the two chains of the therapeutic. Glycosylated and non-glycosylated Knob-Fc-GDF15 peaks annotated. (**b**) Sample control trace (red), a de-glycosylated control sample treated with PNGase F. Blue trace as shown in (**a**).

Supplementary Fig. 3

a

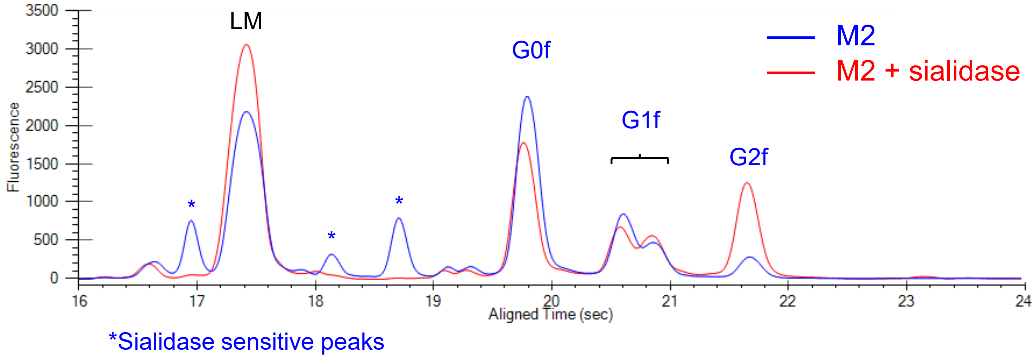

b

GDF15 variant	% Glycan occupancy
Fc-GDF15 WT	-
Mutant 2	98.9%
Mutant 2C	99.2%
Mutant 3	99.7%
Mutant 3B	99.4%

Supplementary Fig. 3. Glycan analysis of in vivo Fc-GDF15 material. (**a**) Representative glycan profiling trace using capillary gel electrophoresis. Blue, Mutant 2 sample. Red, sialidase-treated control. Peaks corresponding to glycan standards annotated as G0f, G1f, and G2f. Asterisks denote sialidase sensitive peaks. LM, lower marker. (**b**) Summary table of engineered site N-glycan occupancy of Fc-GDF15 variants compared to wildtype (WT) control.

Supplementary Fig. 4

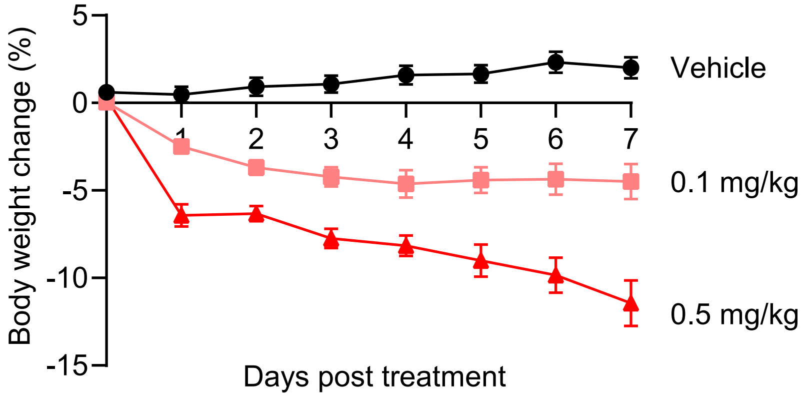

Supplementary Fig. 4. Dose-finding study. Body weight change after a single subcutaneous dose of Fc-GDF15 Mutant 2C at 0.1 and 0.5 mg/kg. n=10 per group. Data are reported as mean ± SEM.

Supplementary Fig. 5

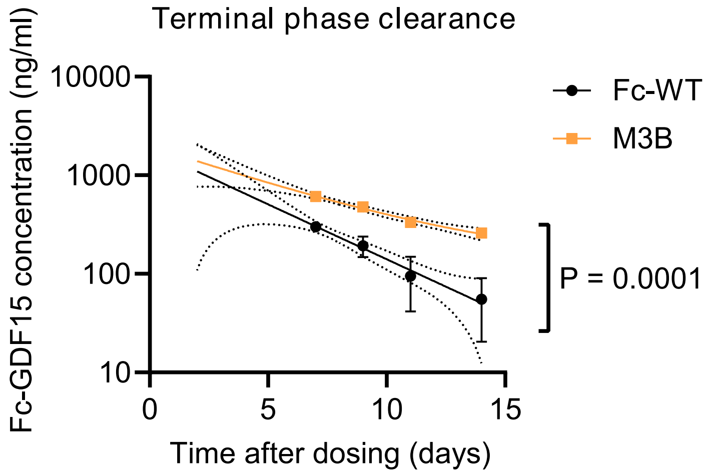

Variant	$T_{1/2}$ (95% CI)
Fc-WT	2.7 days (2.10-3.57)
M3B	5.3 days (4.57-6.23)

Supplementary Fig. 5. Mutant 3B (R4N/G6T/L36R) has extended half-life compared to wildtype Fc-GDF15. Terminal phase clearance in mouse following a single subcutaneous dose of Mutant 3B and wildtype Fc-GDF15 (Fc-WT) control. Half-life reported in days with 95% confidence interval (CI). Dose, 0.1 mg/kg. n=5 per time point. Data are reported as mean ± SD and fit with a single exponential decay model. Dotted lines, 95% CI. Decay rates were statistically compared using an extra sum-of-squares F test (P = 0.0001).

Supplementary Fig. 6

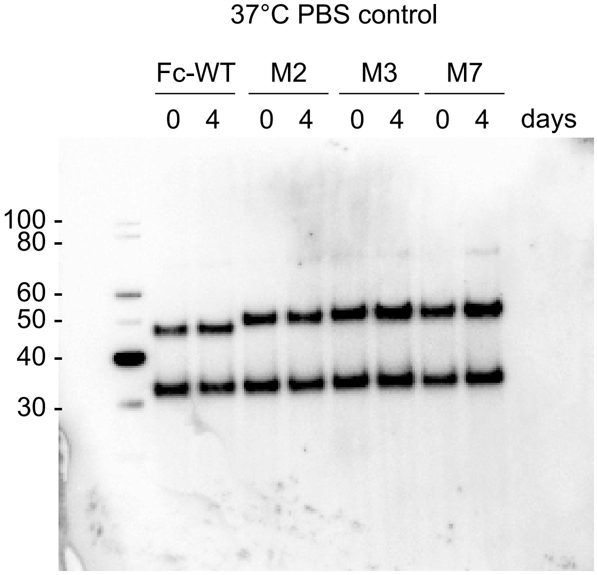

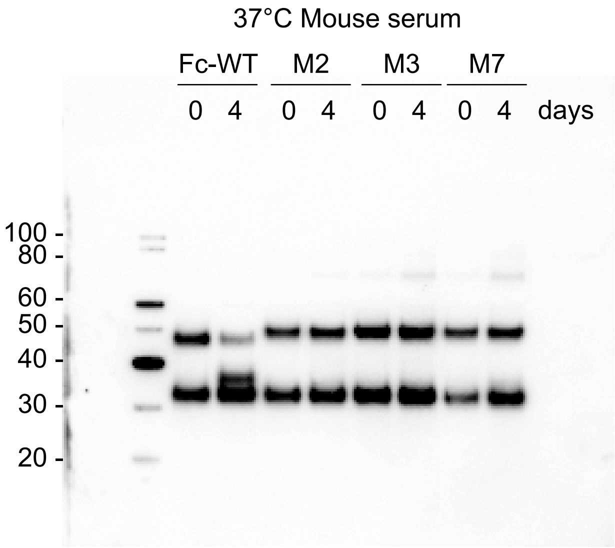

Supplementary Fig. 6. Full scans of Western blots in Figure 2c.

Supplementary Fig. 7

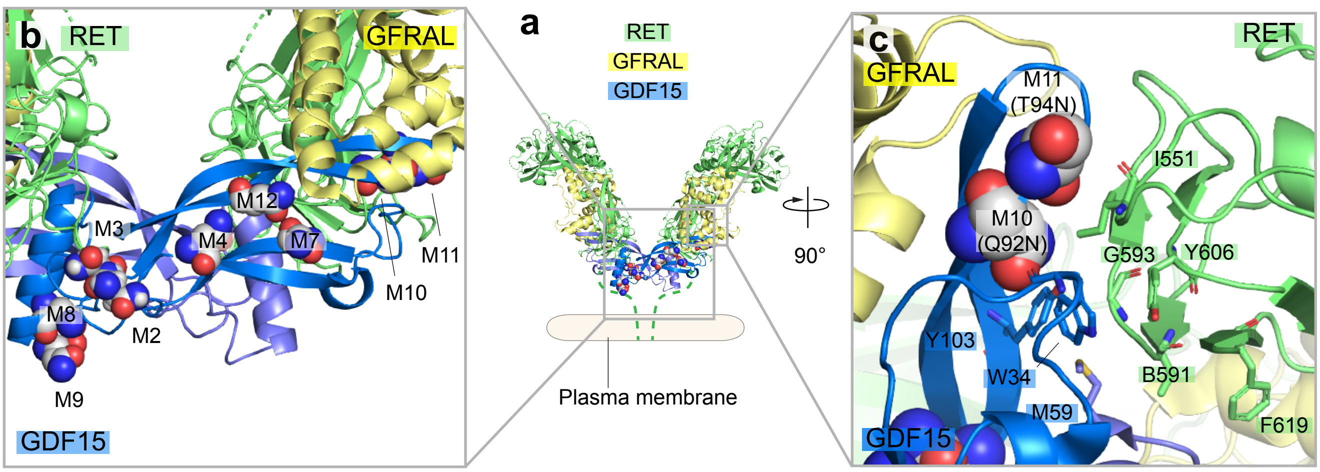

Supplementary Fig. 7. Location of engineered N-glycan sites in GDF15-GFRAL-RET ternary structure. (**a**) Location of engineered N-glycan sites in overall structure of 2:2:2 GDF15 GFRAL RET ternary complex (PDB 6Q2J). Dotted lines indicate the connection from the extracellular domain of RET to its transmembrane region. Adapted from Li et al. [25]. Green, RET. Yellow, GFRAL. Blue, GDF15. Introduced asparagine (N) mutations are shown as spheres. (**b**) Inset showing location of engineered N-glycan sites, shown as spheres. (**c**) Inset showing GDF15-RET interaction site. Mutant 10 (Q92N) and Mutant 11 (T94N) are shown as spheres. W34, Y103, and M59 are involved in RET interaction. Figures rendered using The PyMOL Molecular Graphics System, Version 2.0 Schrödinger, LLC (https://pymol.org/).
